# When Is Forgetting Not Forgetting? A Discursive Analysis of Differences in Forgetting Talk Between Adults With Cystic Fibrosis With Different Levels of Adherence to Nebulizer Treatments

**DOI:** 10.1177/1049732319856580

**Published:** 2019-07-13

**Authors:** Sarah J. Drabble, Alicia O’Cathain, Madelynne A. Arden, Marlene Hutchings, Daniel Beever, Martin Wildman

**Affiliations:** 1 The University of Sheffield, Sheffield, United Kingdom; 2 Sheffield Hallam University, Sheffield, United Kingdom; 3 Sheffield Teaching Hospitals, Sheffield, United Kingdom

**Keywords:** United Kingdom, adherence, cystic fibrosis, memory, chronic conditions, intervention, qualitative, discourse analysis, interviews

## Abstract

Forgetting is often cited as a reason why people struggle to adhere to treatments for chronic conditions. Interventions have tried to improve forgetting behavior using reminders. We used a discursive psychological approach to explore differences in how high and low adherers constructed forgetting their nebulizer treatments for cystic fibrosis. Interviews were conducted with 18 adults from a cystic fibrosis center in the United Kingdom. High adherers constructed forgetting treatments as occasional lapses in automaticity and temporary lapses in memory that they found easy to repair. Low adherers utilized forgetting to normalize more consistent nonadherence to treatments. However, it is important to contextualize forgetting as a discursive resource that helped these participants to negotiate moral discourses around adherence to treatment that reminder interventions cannot address; we therefore recommend a more behavioral, patient-focused, theory-driven approach to intervention development.

## Introduction

Adherence is defined as “the extent to which the patient’s behavior matches agreed recommendations from the prescriber” (Horne et al., 2005, p. 33) and is recognized as a complex behavior that varies between individuals (Kahwati et al., 2016). Low adherence to treatment is a global health problem that has been linked to poorer outcomes such as increased morbidity, mortality, and other costs across a range of clinical conditions (Conn & Ruppar, 2017), including chronic health conditions such as cystic fibrosis (CF).

### Adherence to Nebulizer Treatments in CF

CF is a medical condition affecting around 0.7 people in every 10,000 people in the United States and Europe (Farrell, 2008), including around 10,400 people in the United Kingdom (Cystic Fibrosis Trust, 2012). It is a life-limiting disease and, although life expectancy rates have increased, median survival is projected to be 47 years (Cystic Fibrosis Trust, 2018). CF is a genetic condition characterized by a buildup of sticky mucus that predominantly affects not only the lungs, but also the digestive system and other organs. It is typified by repeated infections of the lower respiratory tract that cause difficulty in breathing, leading to lung damage and ultimately death from respiratory failure.

Medications to improve respiratory function, including mucolytics and antibiotics delivered by nebulizers, have been shown to be effective in reducing exacerbations and preserving lung function (Briesacher et al., 2011; Eakin, Bilderback, Boyle, Mogayzel, & Riekert, 2011; Ryan, Singh, & Dwan, 2011; Smith, Rowbotham, & Regan, 2018; Yang & Montgomery, 2018). However, consistent with other chronic conditions, adherence to treatment is low, with the median rate of nebulizer adherence of adults in clinical practice ranging from 31% to 53% for inhaled antibiotics and from 53% to 79% for inhaled mucolytics (Eakin et al., 2011).

Research has identified a number of barriers and facilitators to adherence in the adult and pediatric population with CF, including to nebulizer treatments. Commonly reported barriers to adherence include treatment burden, practical issues such as collecting prescriptions, and lack of time to do treatments; competing goals including a desire to be “normal”; lack of perceived treatment effectiveness and understanding of treatment recommendations; negative feelings about nebulizers or more general anxiety and depression; social and work demands; personal characteristics; current state of health; and forgetting (Abbott, Havermans, & Hart, 2009; Arden, Drabble, O’Cathain, Hutchings, & Wildman, 2019; Arias-Llorente, García, & Martín, 2011; George et al., 2010; Hogan, Bonney, Brien, Karamy, & Aslani, 2015; Horky, Sherman, & Polvinen, 2014; Lask, 1994; Macdonald et al., 2016; Plummer, Costall, & Torry, 2008; Sawicki, Heller, Demars, & Robinson, 2014).

Reported facilitators of adherence include accessibility and portability of treatment, adherence as part of an individual’s identity and maintaining control, awareness of the importance of treatment, social support, and having a routine (Arden et al., 2019; Arias-Llorente et al., 2011; Foster et al., 2001; George et al., 2010; Hogan et al., 2015; Hoo, Boote, Wildman, Campbell, & Gardner, 2017; Horky et al., 2014; Plummer et al., 2008; Sawicki et al., 2014).

Forgetting to take medication has been identified as a major barrier for both the adult and pediatric population with CF (Abbott et al., 2009; Bregnballe, Schiøtz, Boisen, Pressler, & Thastum, 2011; Dziuban, Saab-Abazeed, Chaudhry, Streetman, & Nasr, 2010; George et al., 2010; Horky et al., 2014; Modi & Quittner, 2006; Owen & John, 2016; Plummer et al., 2008; Sawicki et al., 2014). For example, Sawicki and colleagues (2014) reported that 29% of adolescent participants cited forgetting as the reason for not undertaking their nebulizer treatments. However, two smaller studies in adult patients have started to question the role of forgetting in adherence behavior, suggesting that nebulizer treatments in adults may be so routinized that forgetting is not an issue (Hogan et al., 2015). In a study of the differences in barriers and facilitators to adherence of nebulizer treatments between high and low adherers, Arden et al. (2019) found that forgetting was given as a reason for nonadherence by adults regardless of whether they had high or low adherence, suggesting that addressing forgetting may not be a useful way to improve adherence.

### Moral Issues in Nonadherent Behavior

In the adherence literature, nonadherence has been categorized as intentional or unintentional (Horne et al., 2005). Intentional nonadherence is when people make a conscious, deliberate decision not to adhere to their treatment either some or all of the time and is indicative of low motivation to adhere. Unintentional nonadherence is where people miss treatments inadvertently for reasons out of their control (Gadkari & McHorney, 2012) even though they are motivated to adhere.

The term adherence tries to move away from normative judgments about good (compliant) and bad (noncompliant) behavior, to emphasize the patient’s right to choose whether to follow the prescriber’s recommendations (Horne et al., 2005). However, there is an underlying assumption that the patient *should* follow adherence advice because it is rational to maintain good health, thus creating a normative expectation that patients will adhere to treatment unless there is a good reason not to. Not following this assumption means the patient’s credibility as a good person who is morally adequate (Steele, 1988), and their identity as a “good patient,” may be questioned. In a study of self-management of long-term conditions (stroke, diabetes, and colorectal cancer), Ellis et al. (2017) defined the “good self-manager” as someone who takes treatments within the medical parameters set by the health service. They argued that this definition assumes that the patient chooses to adhere to treatment and has the ability to control any behavioral change to adhere. However, this fails to recognize that taking recommended treatments may not be what individuals want because patients can make rational decisions to not adhere that make sense to them (Donovan & Blake, 1992). Thus, McHorney (2016) argued that nonadherence means that “issues important to the patient were not addressed” (p. 473). Furthermore, behavioral change may be difficult for individuals.

Research suggests that people can be unaware of their reasons for nonadherent behavior (e.g., Wegner, 2004). Navigating assumptions around expectations that patients will do their treatments creates a moral dilemma for the patient: How do they maintain their identity as a good patient if they do not adhere to treatment, even if it is based on what the patient perceives as a perfectly rational decision or something outside of their ability to change?

With regard to forgetting as a reason for nonadherence, forgetting is commonly understood as a lapse in memory that happens occasionally unless there is damage to the memory through accident or ill health (Spear, 2014) and is thus unintentional. However, in a survey of adherence behavior in 24,017 adults with chronic conditions such as asthma or diabetes, Gadkari and McHorney (2012) questioned whether forgetting was really unintentional. They found that forgetting, along with other unintentional nonadherence behaviors, such as carelessness, mediated the relationship between medication beliefs and intentional nonadherence. They suggested that forgetting might instead predict future intentional nonadherence to treatment.

### A Discursive Psychological Approach

A discursive psychological approach focuses on language as action, considering how people utilize language during interactions to construct particular versions or realities, and the functions these constructions serve (Potter & Wetherell, 1987). Language is thus “a tool to get things done” (Potter & Wetherell, 1987, p. 87), for example, to justify a particular action (Pomerantz, 1986) such as adherent or nonadherent behavior.

Although naturally occurring talk is preferred to interviews as the optimal way to consider language in use, McAvoy (2016, p. 103) has argued that “interviews reproduce culturally available discursive resources for making sense of particular meaning-making moments”. Interviews provide a way for researchers to enquire purposely about a particular topic, such as adherence to nebulizer treatments, while allowing talk about forgetting to emerge more naturally during speech, therefore enabling the researcher to examine the function that it serves. Discursive psychology is also interested in how credibility is maintained during talk. In terms of adherence, this includes the moral aspects of adherence behavior—that there is a common understanding that patients should be adhering to treatment—and thus how talking about nonadherence potentially creates the need to address moral judgments and normative expectations about adherent and nonadherent behavior (Bergmann, 1998). Attending to the discursive strategies that speakers utilize to construct their accounts, and particularly the differences between high and low adherers, could enable the functions that forgetting talk served to be made more visible, in particular, how speakers use forgetting talk to justify nonadherence including avoidance of accepting responsibility for their nonadherence.

## Method

We undertook the research reported here as part of a larger study—the Adherence to nebulizer treatments in adults with Cystic Fibrosis (ACtiF) study. ACtiF is a 5-year program to develop and evaluate a theory-based complex intervention to improve nebulizer adherence in adults with CF. The qualitative interview study reported in this article was undertaken at the beginning of the ACtiF program, as the first step in developing an intervention to improve nebulizer adherence, by understanding the barriers and facilitators to nebulizer adherence in adults with CF (Arden et al., 2019). For more information about the intervention and pilot feasibility study, also see the work by Hind et al. (2019).

### Participants

We recruited adults with CF (defined as aged 16 years and above) from a hospital-based CF center in the north of the United Kingdom. We used purposive sampling to approach adults with different objectively measured adherence levels. We also maximized diversity within the sample by approaching adults of different genders, ages, and socioeconomic backgrounds. We approached 21 adults for interview, from whom 20 consented and 18 were interviewed. Participants were mostly male, and well distributed across age groups, adherence levels, and social deprivation quintiles, with the exception of a lack of participants from highly affluent areas. Supplemental Table describes participant characteristics and levels of objective adherence. All participants were White British, which is representative of the CF population in the United Kingdom. Most participants had been diagnosed with CF at birth or shortly after, although two participants had been diagnosed during childhood, and two as adults. Twelve participants were single and three had children. Nine participants worked or studied full-time, two worked or studied part-time, four were not working, and three had caring responsibilities.

### Interviews

We obtained ethical approval from the National Health Service (NHS) research ethics committee (REC) South Central–Hampshire A (14/SC/1455). I (Sarah) arranged and conducted all interviews. I invited participants by letter or email, followed up by a telephone call or emails allowing further discussion before deciding whether to take part. I obtained written informed consent from all participants including that objective adherence data would be discussed during the interview. I conducted interviews between April and August 2015, which took place in participants’ homes or the hospital-based unit depending on their preference.

Each interview followed the same format using a topic guide to help cover all aspects of nebulizer adherence, but leaving the interview open so that the participant could describe their own approach to adherence. I started interviews by asking participants general questions about living with CF and undertaking any treatments before focusing on nebulizer usage. I then introduced adherence graphs displaying the participant’s personalized nebulizer treatment adherence history for the previous 6 months (collected as part of routine treatment in the hospital in numerical form and converted to graphical information for this study) to prompt discussions about times of high and low adherence behavior and what might have caused these (see later for details).

I also used prompts related to the theoretical domains framework (Cane, O’Connor, & Michie, 2012) to explore any other aspects of adherence not already covered. In this latter part of the interview, there were two prompts in the topic guide about forgetting: “How did you remember to use your nebulizer?” and “Is using your nebulizer something that you always remember to do?” However, talk about forgetting occurred naturally throughout the interviews as participants described times when they had struggled with adherence to their nebulizer treatments. Interviews lasted on average 99 min, ranging from 65 to 147 min.

#### Objectively measured adherence

One aspect of the interview was the introduction of objective adherence data regarding nebulizer treatments into the interview. Adherence to nebulizers, like many other treatments, is usually measured via self-report—which has been shown to be inaccurate (Daniels et al., 2011). To measure adherence objectively, we utilized a chipped nebulizer (I-Neb^®^; Philips Respironics) that recorded how much treatment patients had taken compared with the amount of medication prescribed.

In our sample, participants were on a range of nebulized treatments. All were on at least one mucolytic treatment to clear mucous, with most having at least a further two or three preventive antibiotics. A minority of participants were on a separate bronchodilator because most patients mixed this with their antibiotic treatment. The number of daily treatments did not relate to the objective adherence level of our participants as the two lowest adherers had only one daily treatment, and the two highest adherers were taking between two and four daily treatments.

Objective adherence was categorized into high (≥80%), moderate (50.1%-79%), low (25.1%-50%), or very low (≤25%) adherence in the previous 6 months. This was based on prior research that has linked high adherence (≥80%) to better health outcomes, when compared with low adherence (<50%) (Eakin et al., 2011). In addition, we distinguished between very low and low adherence (Hoo, Campbell, et al., 2017). Adherence data were transformed into graphs of adherence in three formats: overall adherence over the past 6 months, days of the week (whereby adherence for each day for the past 6 months was shown), and time of day (where adherence data were shown in 2-hr slots); this was done to help participants identify patterns in their adherence.

### Transcription and Analysis

Interviews were digitally audio-recorded and transcribed verbatim. Maddy and I analyzed the data using framework analysis (Ritchie & Spencer, 1994) to describe facilitators and barriers to adherence; this is reported in a separate article (Arden et al., 2019). During this analysis, I (Sarah) was struck by how both high and low adherers talked about forgetting, and that it seemed to be fulfilling a function within the discussion of adherence. Therefore, I carried out a further discursive analysis on transcript extracts where participants spoke about forgetting as a reason for not doing treatments or having problems remembering their nebulizer treatments, including talk about use of reminders and other cues and prompts.

I retranscribed these extracts using a modified Jeffersonian transcription system (see the appendix). These new transcripts were read and reread and I applied the following questions: (a) How did participants describe and explain forgetting behavior? (b) What functions did these descriptions of forgetting behavior fulfill within this interaction? (c) Did individuals in different adherence categories use forgetting in different ways to explain nonadherence?

I paid attention to patterns of language and aspects of speech such as grammar and word choice, including interactional difficulties signaled by hedging with pauses (Jefferson, 1989), hesitations and repetitions (Buttny, 1993), extreme case formulation (Pomerantz, 1986), and lists of three parts (Jefferson, 1990), which are used to legitimize claims. I also considered modality, which is achieved by using tenses of verbs or adverb; for example, *may/might, could*, and *sometimes* offer less commitment to the truth than *is, has*, or *always* (Fairclough, 2003).

In discussion with Alicia, we noticed patterns in how forgetting was presented by participants with different adherence levels and how this enabled them to avoid talking about other reasons for nonadherence. I used adherence levels to organize the discursive analysis. I then presented analysis of extracts to Alicia; these were discussed in depth, and she challenged my interpretation and analysis in terms of clarity and patterns of speech. A draft of the findings was circulated to the other authors, including a patient representative (Dan), for further challenge and refinement.

## Results

In the results, participants are given a number depending on their adherence level, from the lowest (1) to the highest (18) adherence. Conventionally, discursive analysis would present line-by-line analysis. However, due to the limitations of space, and the range of different ways in which participants used forgetting during adherence talk, we present less detail to show examples of each theme.

In the extracts, “I” refers to me (Sarah) as the interviewer and “P” to the participant. The sample of extracts presented represents the range of functions forgetting served within the interactions in the 18 interviews. In this analysis, we do not in any way want to suggest that patients were deliberately trying to deceive about their adherence. Adherence is behaviorally complex, and there are many reasons why individuals do not adhere, some of which remain unknown to them. However, in talking about adherence and nonadherence, patients are required to negotiate these underlying moral issues and we believe it is important to explore how this happens in conversations about adherence.

### Overview of Forgetting Themes to Justify Nonadherence

Fourteen of our 18 participants cited forgetting as a reason for nonadherence to nebulizer treatments. Of the 18 participants, all but the lowest adherer talked about using different types of reminders to do treatment. We found some interesting ways in which forgetting was utilized by participants. In light of the moral issues identified in the introduction, we recognize that nonadherence was at times difficult for participants to acknowledge. Therefore, we have used the term “admit” to show how participants had to overcome moral issues during the interviews. We present instances where participants admitted nonadherence, times when they occasionally forgot their treatment, strategies to normalize nonadherence, and how well reminders worked to help them remember to do their treatment.

A summary of themes regarding how participants constructed forgetting by adherence level is presented in Table 1. A discussion of each theme with example quotes is presented below.

**Table 1.** A Summary of Themes by Adherence Level.

Constructions of Forgetting	Number of Participants by Adherence Level			
	Very Low *N* = 5	Low *N* = 5	Moderate *N* = 4	High *N* = 4
Admitting nonadherence	1	0	1	1
Occasional lapse in automaticity	0	0	0	3
Temporary lapse in memory	0	0	2	4
Struggling to admit nonadherence	1	1	0	0
Strategies to normalize nonadherence:				
Forgetting as humorous behavior	0	0	1	0
Using socially acceptable justifications for forgetting treatment (e.g., busyness, socializing, tiredness or apathy, and treatment-taking knowledge)	2	3	2	4
Forgetting as avoidance of cystic fibrosis	3	0	0	0
Forgetting as the binary opposite of adherence	0	2	0	0
Forgetting as routinizing nonadherence	3	0	0	0

### Admitting Nonadherence

Admitting nonadherence appeared easier for those with higher adherence, with the exception of one participant who had zero adherence and was open about nonadherence. For example, a very low adherer contested the definition of intentional nonadherence I used as “a conscious decision not to use your nebulizer,” which caused interactional difficulty indicated by the long pauses (Jefferson, 1989). My use of “conscious decision” was an attempt to ask about intentional nonadherence in a different way. The participant questioned the meaning as “plan not to take it anywhere” and I clarified this as “plans not to use it at a particular time because of something” *still* suggesting intentional nonadherence but softening the statement slightly. However, the participant was still unable or unwilling to admit planning not to do their treatment, using “probably forgetting” to explain times of nonadherence as preferable to *admitting (at least consciously)* not to adhere to treatment:

I: are there times when you’ve made like a conscious decision not to use your nebulizer?

P: (2 secs) erm (4 secs) what like sort of plan not to take it anywhere with me?

I: Yeah or plans not to use it at particular time because of something?

P: Not that I’ve planned. I’ve probably forgot (3 secs) to take it. But I’ve not planned not to take it with me. (Very low adherer)

A high adherer distinguished between forgetting (not a choice) and intentional nonadherence (choosing to not adhere), but this required them to own up to this behavior by saying, “I will be honest,” suggesting awareness that nonadherence is not good behavior:

I will be honest, it’s weren’t that I forgot. I just chose not to. (High adherer)

### Forgetting as an Occasional Lapse in Automaticity

Some participants with high adherence described adherence behavior as a habit that was automatic or “hardwired,” therefore making forgetting less likely. For one high adherer, a reduction in prescribed treatment could feel like forgetting because their routine was so automatic that they felt its absence:

P: Just because it’s hardwired isn’t it?

I: (laughs) Whatever it is, is just like-

P: If I come off these many antibiotics, from twice a day, I feel like I’m forgetting something. You know as soon as I finish them if I’m not taking them I start and-

I: What should I be doing?

P: Yeah. (High adherer)

### Forgetting as a Temporary Lapse in Memory

Participants with high adherence also constructed forgetting as a temporary lapse in memory corrected by taking treatment as soon as they remembered, rather than not adhering:

And if I was coming back and then going straight back out and I knew that I needed to take it. Then I just totally forgot. I was out and remembered. (High adherer)

One participant with moderate adherence also constructed forgetting as a temporary lapse that they needed to fix later on. However, remembering the later treatment appeared more effortful than for those with higher adherence, indicated by the use of “it forces you to think” and “you’ve gotta remember”:

It forces you to think, right, now you’ve gotta remember to take it this afternoon because you forgot the morning’s one. (Moderate adherer)

### Strategies to Normalize Nonadherence

Participants showed awareness of normative expectations to do treatments. This created difficulties in admitting to nonadherence, which then needed to be justified in a socially acceptable way. Forgetting was one way participants explained nonadherence and was utilized in a number of different ways: as humorous behavior, as a socially acceptable justification, as avoidance of CF, as the binary opposite of adherence, and as routinizing nonadherence.

#### Forgetting as humorous behavior

In the following example, we all (the interviewer, interviewee, and their wife) laughed about this moderate adherer only taking half their treatment despite the potentially life-limiting consequences of this action. The participant revealed a normative understanding that they should adhere to treatment “I should take it” and that adherence is good behavior “that’s not good.” When the participant used forgetting that morning to explain nonadherence, they laughed and the others in the room joined in. It is funny because after acknowledging that treatment is important, they have been found out and feel the need to explain what has gone wrong. Forgetting as humorous behavior helped to close down the conversation:

That’s not good, I’m only taking it 50 percent of the time, well 55 percent of the time I should take it [wife in background: you haven’t had it yet this morning, you’ve missed this morning ((laughs))], I forgot, yeah ((laughs)). ((All laugh)). (Moderate adherer)

#### Using socially acceptable justifications for forgetting

Both high and low adherers normalized nonadherence by suggesting that simply forgetting was due to a lack of routine, socializing, being too busy, being tired or apathetic, or due to knowledge that treatments should be taken at a number of hours after a previous treatment. At times, forgetting appeared to work as both a synonym for nonadherence and as an explanation for nonadherent behavior that participants did not understand. A very low adherer claimed that socializing led to forgetting, which allowed them to talk about intentional nonadherence in a more socially acceptable way, rather than talking about choosing to go out instead of prioritizing their treatment. The use of “might” suggests this is not a definite course of action (Fairclough, 2003); this was followed by talking about repairing this behavior by playing “catch up” the following day to persuade me that this is not common behavior:

Sometimes, if then someone says “oh let’s go out” you know I might then, that might be the time I might forget my evening one. And it just never comes back to mind until sort of the next day and I think “oh I’ve actually missed my evening dose the night before” but then you know I’m sort of trying to play catch up the next day. (Very low adherer)

Some participants constructed forgetting as being too busy to adhere. In the following extract, talk of forgetting and a “busy life” helped the participant to normalize their lack of treatment-taking routine. This moderate adherer started this extract talking about watching a popular television program, Britain’s Got Talent, as “just busy life.” However, perhaps realizing that this excuse needed justifying further, they added having “other things on” or going out, which created a three-part list that gave more plausibility to the justification (Jefferson, 1990). In a later part of the extract, the participant mentions forgetting, in addition to a busy life or getting into a “regime,” so the listener is unclear about whether forgetting is due to a busy life or in addition to a busy life. The description “regimented regime” implies that routines offer a strictly controlled, unappealing alternative to nonadherence, and the listener is left unclear as to whether the participant is unable to establish a routine in the context of a busy life, finds routines unappealing, or does not fully understand their nonadherence behavior:

Oh it’s just, I don’t know, it’s just busy life, you just, we’d be sat watching, I don’t know, Britain’s Got Talent (.) “ah I didn’t take me! Ah!” and that’s how it goes, you just forget or you’ve, you’ve got other things on, or you’ve gone out and, so in terms of not doing your treatment or missing stuff it’s purely on a basis where you’ve, you’ve got a busy life or you’ve forgot or you can’t get into a regimented (.) regime of taking stuff. (Moderate adherer)

A high adherer also admitted nonadherence by presenting a moral dilemma between two socially unacceptable courses of action; nonadherence (forgetting) in some situations is reasonable “you just can’t” if it represents a temporary lapse that cannot be repaired without committing a more socially unacceptable action “you are not just going to come home from someone’s birthday to do your medication”:

There is just going to be times when you just can’t, like it might be someone’s birthday, and you might have forgot to do it. And you are not just going to come home from someone’s birthday to do your medication. (High adherer)

#### Forgetting as avoidance of CF

Some participants constructed nebulizer treatments and prompts to do treatments as unwanted reminders of CF. Forgetting worked to persuade the listener that it is unacceptable to expect someone to think about CF all of the time; therefore, it is acceptable to forget your treatment. This very low adherer described a period of not adhering to treatment by putting off treatment at different time points until they forgot to do it, indicated by “it will completely slip my mind and I just forget to do it altogether.” The use of forgetting persuades the listener that the nonadherent behavior is not a choice but a series of smaller decisions that add up to forgetting, rather than intentional nonadherence. However, I called into question the plausibility of this statement and the participant gives another reason for nonadherence; that they want to be “normal,” more like “someone my age.” Adherence is thus presented as a barrier to normality that justifies the intentional nonadherence in a plausible way:

At the minute it’s just like out the window. Like I’ll say “ah I’ll do that in the morning.” Get up in the morning, and I’ll find something else to do, and it won’t be till later on in the day and I’m like “oh I should have done that, oh I’ll do it later when I get in then.” Get home later and it will completely slip my mind and I just forget to do it altogether.

I-Ok, do you think that’s erm, an intentional thing or?

P-I think it’s more like a blocking and like I put out my mind. Like I say if I block it, it isn’t there sort of thing [yeah] it’s the way it is

I-How does it help you if you block it?

P-Because like, if I don’t, if I block it, I don’t think about it so it’s not there sort of thing I feel. So I can, I don’t know how to say, not be normal, but more of someone my age sort of thing. (Very low adherer)

#### Forgetting as the binary opposite of adherence

A low adherer constructed adherence behavior as “easy” and “simple”; you either remember to do your treatment or you forget to do it. This construction normalized nonadherence, making it harder to open up the conversation about reasons for nonadherence:

I just I either remember or I don’t, it’s as easy, it’s as simple as that. (Low adherer)

Another low adherer constructed forgetting as the binary opposite of perfect adherence, which allowed the participant to normalize nonadherence because no one can be perfect all the time. After openly admitting to “times when you don’t do” treatment, the explanation for this nonadherence was contrasted with doing treatment 100% of the time, which was described as “idealistic,” “perfect” behavior that is essentially unachievable. The addition of “never,” an extreme case formulation, legitimizes never forgetting as unrealistic (Pomerantz, 1986):

I think there are times when you don’t do them. I think you would be very err idealistic to just say “oh yes I’m perfect and I do them all the time and I never forget.” (Low adherer)

#### Forgetting as routinizing nonadherence

Other participants with low adherence constructed many smaller instances of forgetting that added up to a period of low adherence to normalize nonadherence. In the following extract, the participant justified why they had not adhered while maintaining their identity as a good patient who is improving their adherence. Participants initially constructed forgetting as routinized examples of occasional nonadherence “sometimes it can lead to sort of forgetting a bit more often.” However, to maintain their identity as a good patient, the participant suggested that recently forgetting had become “known,” “more conscious,” and had improved “trying to find more ideas of how to prevent the forgetfulness.” Thus, they are able to maintain their identity as a good patient who is trying to improve their adherence in the context of someone who is not currently adhering:

I think the consequences of that [missing your nebulizer] is you know sometimes it can lead to sort of forgetting a bit more often but I think you know the times that I forgot recently, I’ve sort of known about it and I’ve been more conscious about it and I think finding more ideas trying to find more ideas of how to prevent the forgetfulness. (Very low adherer)

In the following extract, the participant suggested that making smaller conscious decisions to put off treatment leads to forgetting, “and then I forget,” rather than making an intentional decision not to do treatment. This suggestion is made more plausible by suggesting that it is an occasional disruption that only occurs at weekends to their usual “regimented routine” that the listener assumes happens during the week. It is only my knowledge of the objectively measured very low adherence of this participant that calls this into question:

But also on a weekend if I don’t get up and we’re not going out there’s not that regimented routine so I just think “oh I’ll do it in a bit” and then a bit comes and I think I’ll do it in a bit and it just doesn’t get done and then I forget. (Very low adherer)

### Using Reminders to Address Forgetting

Thirteen of the 18 participants talked about currently or having in the past used some form of reminder to do their treatment, for example, an alarm, chart, or visual prompt such as leaving their nebulizer where they could see it. Of those who talked about reminders, five participants, four of whom had the highest adherence, described having a strong routine, which rendered reminders redundant. However, they did think that reminders could be useful at times when treatments changed, and two of them had previously used visual prompts. In contrast, participants with very low to moderate adherence found reminders had limited usefulness, actively ignored alarms on phones, and described them as “nagging” (low adherer) or annoying:

My psychiatrist once mentioned [reminders], tried that and set alarms on my phone. And my phone just nearly, on a bad day, ended up through the window. Shut up. (Very low adherer)

Alternatively, reminders were just another thing to be forgotten:

But that’s just something else to remember to tick [] and I always forgot to do it so it’s like “oh.” (Low adherer)

Some participants used other people, such as parents, to remind them to do their treatment, suggesting a lack of self-efficacy. For example, a very low adherer appeared to shift responsibility to their parent, signaled in the quote below by “you’re going to have to” and “tell me then it will remind me”:

And I said to my mum [] you’re going to have to tell me “don’t forget your nebulizer” you know “just remind me, tell me” then it will remind me to sort of keep going with it. (Very low adherer)

## Discussion

Using a discursive psychological approach, we found that forgetting talk fulfilled discursive functions related to both intentional and unintentional nonadherence, rather than only the expected unintentional nonadherence (Gadkari & McHorney, 2012).

### Low Versus High Adherence

In this study, we adopted a novel approach, using chipped nebulizer data to compare how talk about forgetting to do your treatment differed between high and low adherers. Higher adherers appeared to have a clearer understanding of their own behavior, creating a conceptual framework to explain occasional and temporary lapses of memory and routines that could be repaired, drawing on the common understanding that memory only fails occasionally and will be rectified later (Spear, 2014).

Recent research has suggested that reminder interventions may not always be an effective strategy to improve adherence (e.g., Choudhry et al., 2017; Kahwati et al., 2016). We found that reminders were perceived as unnecessary by high adherers, perhaps due to their well-established routines, and unhelpful and easy to ignore by lower adherers, although (visual) cues such as leaving equipment where it is visible seemed to be useful at least when establishing adherence habits.

Our study also adds to understanding of forgetting as a reason for nonadherence (e.g., Gadkari & McHorney, 2012; McHorney, 2016) by identifying how participants with very low adherence normalized nonadherence by routinizing smaller, occasional lapses into a longer period described as forgetting, rather than nonadherence. Low adherers also contrasted forgetting with perfect adherence to persuade the listener that some adherence behavior was essentially unachievable, shutting down other explanations for nonadherence behavior that they found difficult to explain or did not understand.

### Forgetting as a Way to Address Moral Dilemmas in Admitting Nonadherence

The adherence literature has moved from talking about compliance, incorporating normative expectations about bad and good behavior, to adherence, in which the patient decides whether to follow adherence advice (Donovan & Blake, 1992; for example, Horne et al., 2005). However, in this study, both the interviewer and participants appeared to share the expectation that participants *should* adhere to treatment, that this represented good behavior, and that any nonadherence needed to be justified.

Admitting nonadherence potentially threatened participants’ identities as a “good person” (Steele, 1988) and a “good patient or self-manager” who “takes treatments within medical parameters” (Ellis et al., 2017). The concept of the good patient draws on Parsons’s (1975) sociological concept of the sick role in which a patient has an obligation to get better or become healthier (Burnham, 2014). Forgetting was one way in which participants could explain nonadherent behavior, while maintaining the identity of the patient who is doing what they can to get better except in situations in which it is not possible. Although, more recently, sociologists have moved away from the concept of the sick role to the patient having control of their treatment (Burnham, 2014; Donovan & Blake, 1992), in our study, nonadherence still needed to be addressed within verbal interactions. Thus, forgetting became a discursive resource to help participants negotiate some of the moral discourses around adhering to treatment (Murdoch, Salter, Poland, & Cross, 2015).

Participants with very low adherence used avoidance of thinking about having CF as a reason for forgetting. Avoidance has been identified by others as a way of coping with CF (Abbott, Dodd, Gee, & Webb, 2001) and has been associated with higher self-esteem, lower alienation, and discomfort in young adults with CF and rated by physicians as an effective coping strategy (Moise, Drotar, Doershuk, & Stern, 1987). In health care consultations, there is a recognized power imbalance (Joseph-Williams, Elwyn, & Edwards, 2014). Strategies that normalize nonadherence, similar to those found in our study, could deflect attention away from a more challenging conversation about nonadherence, allowing the patient to regain some control of the interaction (Conrad, 1985; Herrera, Moncada, & Defey, 2017). Forgetting talk may offer patients a way to avoid addressing reasons for nonadherence that could bring them into conflict with health professionals who advocate treatment adherence, while helping them to maintain psychological well-being (Arias-Llorente et al., 2011).

### Limitations

There are some limitations to this study. First, the sample was from a single center that may be different from other centers. This center had started to use measurement of objective adherence to medication within clinical consultations, and motivational interviewing to address nonadherence, at the time this study was undertaken. Motivational interviewing is an approach to interactions in which ambivalence to behavior change is explored and resolved in a nonconfrontational manner (Rollnick & Miller, 1995). The approach of the center may have affected patient views of adherence by making them more positive or negative toward adherence, or more aware of adherence, but the interviewer had no sense in the interviews that this had occurred. In addition, motivational interviewing training is not unique to this center and may occur in many CF centers to some extent.

Second, participants were made aware that they would be seeing and discussing graphs of their adherence data as part of this interview when agreeing to participate. Knowing this may have encouraged participation by those more open to talking about nonadherence but also created a situation in which (non)adherence was the topic of conversation and needed to be justified (Murdoch et al., 2015).

Third, the sample had more male participants than the CF population. We aimed to get a balance of males and females and do not know why this gender imbalance occurred. It is possible that our findings relate more to men than to women; however, all of the issues identified appeared in the small number of females in the study, as well as the males.

### Implications

Forgetting is often cited as a barrier to adherence in both CF (e.g., Abbott et al., 2009; Bregnballe et al., 2011; Dziuban et al., 2010; George et al., 2010; Horky et al., 2014; Kahwati et al., 2016; Modi & Quittner, 2006; O’Toole et al., 2019; Owen & John, 2016; Plummer et al., 2008; Sawicki et al., 2014) and other chronic conditions (Gadkari & McHorney, 2012; Horne et al., 2005; Ingersoll & Cohen, 2008; Mills et al., 2006; Rathbone et al., 2017). We do not claim to have uncovered all the different ways in which forgetting is utilized to account for nonadherence of nebulizer treatments in CF (Morse, 2015). However, we found instances where low adherers used forgetting to describe intentional nonadherence, which has implications for research and practice.

Several measures of subjective adherence make the assumption that forgetting is unintentional, for example, the five-item Medication Adherence Report Scale (MARS-5; Horne & Hankins, 2004) and Morisky Medication Adherence Scale (MMAS; Morisky, Green, & Levine, 1986). In addition, interventions to improve adherence to medication in people with chronic conditions, that only classify forgetting as unintentional nonadherence, will tend to focus on simple solutions that bring adherence back to the forefront of patients’ minds, such as email or text reminders to take medication. This focus may lead to interventions being developed to help participants avoid forgetting that may not be effective (e.g., Choudhry et al., 2017).

We suggest that for some types of treatment for some conditions, forgetting can mean intentional nonadherence (Gadkari & McHorney, 2012); therefore, more complex, behaviorally orientated, psychosocial approaches to increasing adherence may be needed (Conn & Ruppar, 2017; Kronish & Moise, 2017; Molloy & O’Carroll, 2017).

Our findings also have implications for health care practitioners discussing adherence to treatment with patients. Even when patients talk about forgetting treatment, we suggest that health care practitioners should be open to other possible explanations for nonadherence that patients may feel unable to express openly, or have difficulty identifying, and that there may be aspects other than forgetting that patients use to avoid talking about, or to justify, nonadherence.

Rather than relying on self-reported adherence that may be inaccurate (Daniels et al., 2011), the use of objective adherence data from chipped nebulizers can help to open up patient-led discussions about adherence behavior. This approach recognizes that both patients and staff may not know how much someone adheres, or understand why they do or do not adhere (Wegner, 2004), and that the best intentions to adhere can be unstable and easily derailed by contextual factors (Arden et al., 2019). It is important that conversations about adherence are approached in a nonthreatening way, for example, using a motivational interviewing approach (Rollnick & Miller, 1995). Aspects of motivational interviewing can be used as an accompaniment to other behavior change strategies (Kahwati et al., 2016) that take a more complex, theory-driven approach to help patients address difficult behavioral change by addressing aspects such as habit formation (Conn & Ruppar, 2017), emotions, and relationships (O’Toole et al., 2019).

## Conclusion

A discursive psychological approach found that differences between how high and low adherers of CF nebulizer treatments used forgetting to justify nonadherence to nebulizer treatments. Forgetting is usually understood as unintentional nonadherence; however, forgetting became a discursive resource to help participants negotiate some of the moral discourses around adhering to treatment.

## Supplemental Material

supplemental_material – Supplemental material for When Is Forgetting Not Forgetting? A Discursive Analysis of Differences in Forgetting Talk Between Adults With Cystic Fibrosis With Different Levels of Adherence to Nebulizer Treatments Click here for additional data file. Supplemental material, supplemental_material for When Is Forgetting Not Forgetting? A Discursive Analysis of Differences in Forgetting Talk Between Adults With Cystic Fibrosis With Different Levels of Adherence to Nebulizer Treatments by Sarah J. Drabble, Alicia O’Cathain, Madelynne A. Arden, Marlene Hutchings, Daniel Beever and Martin Wildman in Qualitative Health Research

## Appendix

**Appendix:** Transcription notation.

Notation	Description
Word.	A completing intonation (not necessarily a grammatical full stop)
Word,	A continuing intonation
Word	Emphasis by the speaker on a word or part of a word
Wo-	Abrupt termination or word or sound
Wo’d	Letters omitted from words or phrases
“Word”	Reported speech
(.)	Brief pause in the flow of speech
(2 secs)	Longer pause, showing the time in seconds of the pause
Don’t you [mm] I mean	Bold text in square brackets show interviewer speech which did not break the flow of the interview
((interviewee laughs))	Text in double parentheses and italicized refers to notes about how something was said or to something that happened during the interview
[]	Omitted speech
[text]	Clarificatory information from transcription
(. . .)	Inaudible speech
