# Supplementary material for: When Is Forgetting Not Forgetting? A Discursive Analysis of Differences in Forgetting Talk Between Adults With Cystic Fibrosis With Different Levels of Adherence to Nebulizer Treatments
Source: Qual Health Res. 2019 Jul 13;29(14):2119–31. doi: 10.1177/1049732319856580 (PMC7322938; doi:10.1177/1049732319856580)
Supplement: supplemental_material – Supplemental material for When Is Forgetting Not Forgetting? A Discursive Analysis of Differences in Forgetting Talk Between Adults With Cystic Fibrosis With Different Levels of Adherence to Nebulizer Treatments [file supplemental_material.pdf]

Table 1: Characteristics of interview participants

Characteristic	Group (no of participants in sample)
Gender	Male (13) Female (5)
Age, in years	16-18[a] (4)<br>20-25<br>26-30 (8)<br>31-39 (4)<br>40 and over (2)
Marital status	Single (12) Married or co-habiting (6)
Index of Multiple Deprivation quintile[b]	1 (0) most affluent<br>2 (4)<br>3 (5)<br>4 (7)<br>5 (2) most deprived
Mean objective adherence over the previous six months[c]	Zero or very low (5)<br>Low (5)<br>Moderate (4)<br>High (4)

a) Patients transition from the paediatric to adult CF service from the age of 16 so are considered adult for the purposes of this study.
b) Index of Multiple Deprivation by postcode (Ministry of Housing Communities & Local Government, 2015). The Index measures relative deprivation by UK postal code stratifying into 5 quintiles where 1 is the most affluent and 5 is the most deprived. By entering a postal code it is possible to get an indication of the deprivation level where an individual lives.
c) Mean unadjusted objective adherence over previous six months measured from chipped i-neb nebulisers: High (≥80%), moderate (50.1-79%), low (25.1-50%), very low (≤25%) (Eakin et al., 2011; Hoo, Campbell, Curley, & Wildman, 2017)
